# Transcriptome profiling of Arabidopsis *slac1-3* mutant reveals compensatory alterations in gene expression underlying defective stomatal closure

**DOI:** 10.3389/fpls.2022.987606

**Published:** 2022-09-20

**Authors:** Zheng Wang, Yinghui Ouyang, Huimin Ren, Shuo Wang, Dandan Xu, Yirui Xin, Jamshaid Hussain, Guoning Qi

**Affiliations:** ^1^State Key Laboratory of Subtropical Silviculture, School of Forestry and Biotechnology, Zhejiang A&F University, Hangzhou, Zhejiang, China; ^2^Department of Biotechnology, COMSATS University Islamabad, Abbottabad, Pakistan

**Keywords:** drought stress, abscisic acid, stomata, anion channel, transcriptome

## Abstract

Plants adjust their stomatal aperture for regulating CO_2_ uptake and transpiration. S-type anion channel SLAC1 (slow anion channel-associated 1) is required for stomatal closure in response to various stimuli such as abscisic acid, CO_2_, and light/dark transitions etc. Arabidopsis *slac1* mutants exhibited defects in stimulus-induced stomatal closure, reduced sensitivity to darkness, and faster water loss from detached leaves. The global transcriptomic response of a plant with defective stimuli-induced stomatal closure (particularly because of defects in SLAC1) remains to be explored. In the current research we attempted to address the same biological question by comparing the global transcriptomic changes in Arabidopsis *slac1-3* mutant and wild-type (WT) under dark, and dehydration stress, using RNA-sequencing. Abscisic acid (ABA)- and dark-induced stomatal closure was defective in Arabidopsis *slac1-3* mutants, consequently the mutants had cooler leaf temperature than WT. Next, we determined the transcriptomic response of the *slac1-3* mutant and WT under dark and dehydration stress. Under dehydration stress, the molecular response of *slac1-3* mutant was clearly distinct from WT; the number of differentially expressed genes (DEGs) was significantly higher in mutant than WT. Dehydration induced DEGs in mutant were related to hormone signaling pathways, and biotic and abiotic stress response. Although, overall number of DEGs in both genotypes was not different under dark, however, the expression pattern was very much distinct; whereas majority of DEGs in WT were found to be downregulated, in *slac1-3* majority were upregulated under dark. Further, a set 262 DEGs was identified with opposite expression pattern between WT and mutant under light–darkness transition. Amongst these, DEGs belonging to stress hormone pathways, and biotic and abiotic stress response were over-represented. To sum up, we have reported gene expression reprogramming underlying *slac1-3* mutation and resultantly defective stomatal closure in Arabidopsis. Moreover, the induction of biotic and abiotic response in mutant under dehydration and darkness could be suggestive of the role of stomata as a switch in triggering these responses. To summarize, the data presented here provides useful insights into the gene expression reprogramming underlying *slac1-3* mutation and resultant defects in stomatal closure.

## Introduction

Stomatal pores are surrounded by a pair of guard cells. The emergence of latter is considered as a major landmark in the land plants’ evolution and adaptation to harsh environmental conditions (Umezawa et al., 2010). Stomata open and close in response to diverse stimuli and are involved in efficiently taking up CO_2_ for photosynthesis and regulating water loss through transpiration (Jezek and Blatt, 2017).

The opening and closing of the stomatal aperture are brought about by changes in turgor pressure through ion and water channel proteins present in the guard cell plasma membrane (Schroeder et al., 2001; Kim et al., 2010; Engineer et al., 2016). Stomatal movements are tightly regulated under the influence of environmental stimuli and endogenous factors, such as light, water status, carbon dioxide (CO_2_), ABA, calcium (Ca^2+^), and reactive oxygen species (ROS; Schroeder et al., 2001; Young et al., 2006).

It is now well established that light-induced stomatal opening is regulated by plasma membrane inward-rectifying K^+^ channels while stomatal closure involves outward K^+^ channels and anion channels. When plants are challenged with stressful conditions, the activation of guard cell anion channels becomes a key step in inducing stomatal closure. Different signaling components including second messengers, phospholipases, protein kinases, and phosphatases are involved in regulation of stomatal movements. Moreover, stomatal opening and closure are also governed by changes in gene expression.

Abscisic acid- and drought stress induce anion efflux through anion channels, causing membrane depolarization in guard cells. One of the main guard cell anion channels SLAC1 (slow anion channel-associated 1) was identified as an S-type anion channel during the screenings for ozone-sensitive and CO_2_ insensitive mutants (Negi et al., 2008; Vahisalu et al., 2008). SLAC1 is required for stomatal closure in response to various stimuli such as abscisic acid, CO_2_, light/dark transitions, ozone, Ca^2+^ ions, humidity change, nitric oxide, and hydrogen peroxide. Localized in plasma membrane of guard cells, SLAC1 constitutes a trimer in which each subunit forms a channel pore independently (Chen et al., 2010). SLAC1 is a highly conserved S-type anion channel in diverse plant species. SLAC1 homologues have been identified in various species ranging from green algae to higher plants (Hedrich and Geiger, 2017; Ren et al., 2021). Rice *slac1* mutants showed higher stomatal conductance and photosynthetic rate under well-watered conditions (Kusumi et al., 2012). In maize, ZmSLAC1 is involved in stomatal closure mainly by mediating the nitrate efflux (Qi et al., 2018).GhSLAC1 is an essential element for stomatal closure in response to drought in cotton (Ren et al., 2021).In Arabidopsis, five members of SLAC/SLAH family have been reported. Among those, AtSLAC1 and AtSLAH3 are responsible for chloride and nitrate efflux across guard cell plasma membrane (Negi et al., 2008; Vahisalu et al., 2008; Meyer et al., 2010; Qi et al., 2018).

Drought stress triggers increase in ABA which is an important stimulus for SLAC1 activation. After being bound by ABA, PYR/PYL/RCAR receptors interact with PP2C phosphatases and, thereby, previously inhibited Open stomata 1 (OST1) kinase is released which, through phosphorylation, activates SLAC1 channel (Murata et al., 2001; Mustilli et al., 2002; Yoshida et al., 2006; Geiger et al., 2009; Lee et al., 2009; Nishimura et al., 2010; Brandt et al., 2012). This leads to anion efflux from guard cells and subsequent stomatal closure. Besides OST1, other kinases like Leucine-rich repeat kinase (LRR-RLK), Calcium-dependent protein kinases (CPK/CDPK), Mitogen-activated protein kinase (MPK), and Calcineurin-B like protein (CBL)-CBL-interacting protein kinase (CIPK) are also involved in SLAC1 regulation (Mori et al., 2006; Cheong et al., 2007; Jammes et al., 2009; Geiger et al., 2010; Brandt et al., 2012; Scherzer et al., 2012; Maierhofer et al., 2014; Khokon et al., 2015; Prodhan et al., 2018). Moreover, few other stimuli like salicylic acid (SA), methy1 jasmonate (MeJA), and bacterial invasion also trigger stomatal closure *via* SLAC1 (Melotto et al., 2008; Khokon et al., 2011; Yan et al., 2015). In the light of above-cited literature, SLAC1 seems to be a converging point for different stomata closure pathways.

In Arabidopsis, loss-of-function mutations in SLAC1 result in strong impairment of S-type anion channel activity and inhibition of stimulus-induced stomatal closure (Negi et al., 2008; Vahisalu et al., 2008). The *slac1* mutant exhibited reduced sensitivity to darkness. Moreover, higher CO_2_-induced increase in leaf temperature was also inhibited in *slac1-2* mutant. The detached leaves of *slac1* mutants lost water significantly faster than WT, pointing to defects in transpiration regulation under drought stress (Negi et al., 2008; Vahisalu et al., 2008). Mutations in *slac1* also unexpectedly affected the stomatal opening induced by light, low CO_2_, and higher air humidity. This phenotype was found to be associated with higher concentration of resting cytosolic Ca^2+^ and drastically low activity of inward K^+^ channels in *slac1* guard cells (Laanemets et al., 2013).

Although handful literature is available on SLAC1 regulation mechanism, however, the effects of *slac1* mutation, and resultant defects in stomatal closure, on global gene expression need to be explored yet. In current study, we have attempted to answer this question by determining global transcriptomic changes in Arabidopsis *slac1-3* mutant under dark and dehydration stress, by using RNA-sequencing.

## Materials and methods

### Plant materials and growth conditions

The wild-type (WT) Arabidopsis Columbia ecotype was used for the experiments unless otherwise indicated. In all the experiments Arabidopsis *slac1-3* mutant was used. Seeds were surface sterilized with Plant Preservative Mixture (PPM) by keeping at 4°C for 2 days. The seeds were then placed on 1/2 strength Murashige and Skoog (MS) medium (pH 5.7–5.8), containing 1% sucrose and 0.8% agar, and the plates were kept in incubator at 22°C with 12:12 h, light:dark cycle settings for 2 weeks. The seedlings were then transplanted into the soil. Plants were grown at 12:12 h, light:dark, 23:18°C, and 60:70% humidity cycle.

### Plant treatments for RNA sequencing experiment

The aboveground part of WT and mutant plants was cut out at 2 pm (Beijing time) for control. For dark treatment, the material was collected after 2 h of darkness in the greenhouse. For dehydration stress, the aboveground part of the plant was cut off and harvested after 25 min of water loss under natural conditions, as previously reported (Zotova et al., 2018). Samples were immediately frozen in liquid nitrogen and stored at −80°C and then subjected to Illumina sequencing. The experiment was performed in three biological replicates with each replicate consisting of three seedlings.

### Identification of differentially expressed genes and functional enrichment analysis

Bioinformatic analysis was performed using the OmicStudio tools at https://www.omicstudio.cn/tool. The DEGs were identified based on |log2 fold-change| ≥ 1 and value of *p* < 0.05. The genes with expression level greater than 5 were retained. Venn and GO enrichment analysis were also performed using OmicShare Tools.^1^ The heatmaps were drawn by TBtools (Chen et al., 2020).

### Stomatal movement experiment

Fresh plant leaves from 3 weeks old plants were collected at about 2 pm (Beijing time) and placed in stomatal opening buffer (50 μM CaCl_2_, 5 mM KCl, 10 mM MES-Tris, and pH 6.15). The leaves were soaked in buffer with the back side up and exposed to light (200–250 μmol m^−2^ s^−1^) for 2 h to fully open their stomata. Then, the lower epidermis of the leaves was peeled off to make temporary pieces. These were then observed under Zeiss microscope and pictures were taken (as control). For dark treatment, the samples were collected after shading the plants for 2 h. For ABA treatment, 200 μM ABA was sprayed on the plant leaves and the samples of lower epidermis were collected after 25 min. Three replicates were included for each treatment, and at least 30 stomata were measured in each group. The width of stomatal aperture was measured from the images with the help of ImageJ.

### Thermal imaging experiment

The plants (Col-0 and *slac1-3* mutants) were grown for 6 weeks under normal conditions. For dark treatment, the plants were grown without light for 30 min. Plants grown under normal light conditions were used as controls. The aboveground part of the plants was cut and placed on white paper for taking pictures immediately. Three seedlings were observed under each condition. The images were captured with HIKVISION H10 thermal Imager.

## Results

### The dark- and ABA-induced stomatal closure was defective in Arabidopsis *slac1-3* mutants

**Figure 1:**
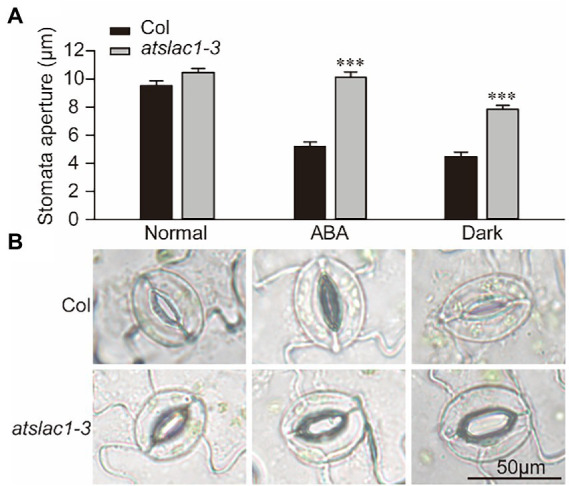
Arabidopsis *slac1-3* mutants are insensitive to abscisic acid (ABA) and darkness. **(A)** Graphical presentation of stomatal aperture under ABA, dark treatment, or light. **(B)** Representative images of stomatal aperture in WT and *slac1-3* mutants under ABA, dark, or light. Three replicates were included for each treatment, and at least 30 stomata were measured in each group. Bars represent mean ± S.E. *** Denotes statistically significant difference (*t*-test *p* < 0.01).

We determined the response of stomata to ABA and dark in epidermal peels of the WT and *slac1-3* mutants of *Arabidopsis thaliana.* Under normal conditions, the stomatal aperture of *slac1-3* mutant was slightly larger than that of WT (Figures 1A,B). When treated with ABA or dark, the stomatal aperture in WT significantly decreased; being about 50% of that of untreated plants. In contrast to this, under ABA treatment, stomatal aperture in *slac1-3* mutant was not significantly different than its untreated control, showing a nearly complete insensitivity to ABA. When shifted from light to dark, although the stomatal aperture in *slac1-3* mutants decreased compared with its non-treated counterpart, however, the extent of stomatal closure was still significantly less than dark-treated WT. These data demonstrate that ABA- and dark-induced stomatal closure is defective in *slac1-3* mutant.

### Arabidopsis *slac1-3* mutation resulted in lower leaf temperature

**Figure 2:**
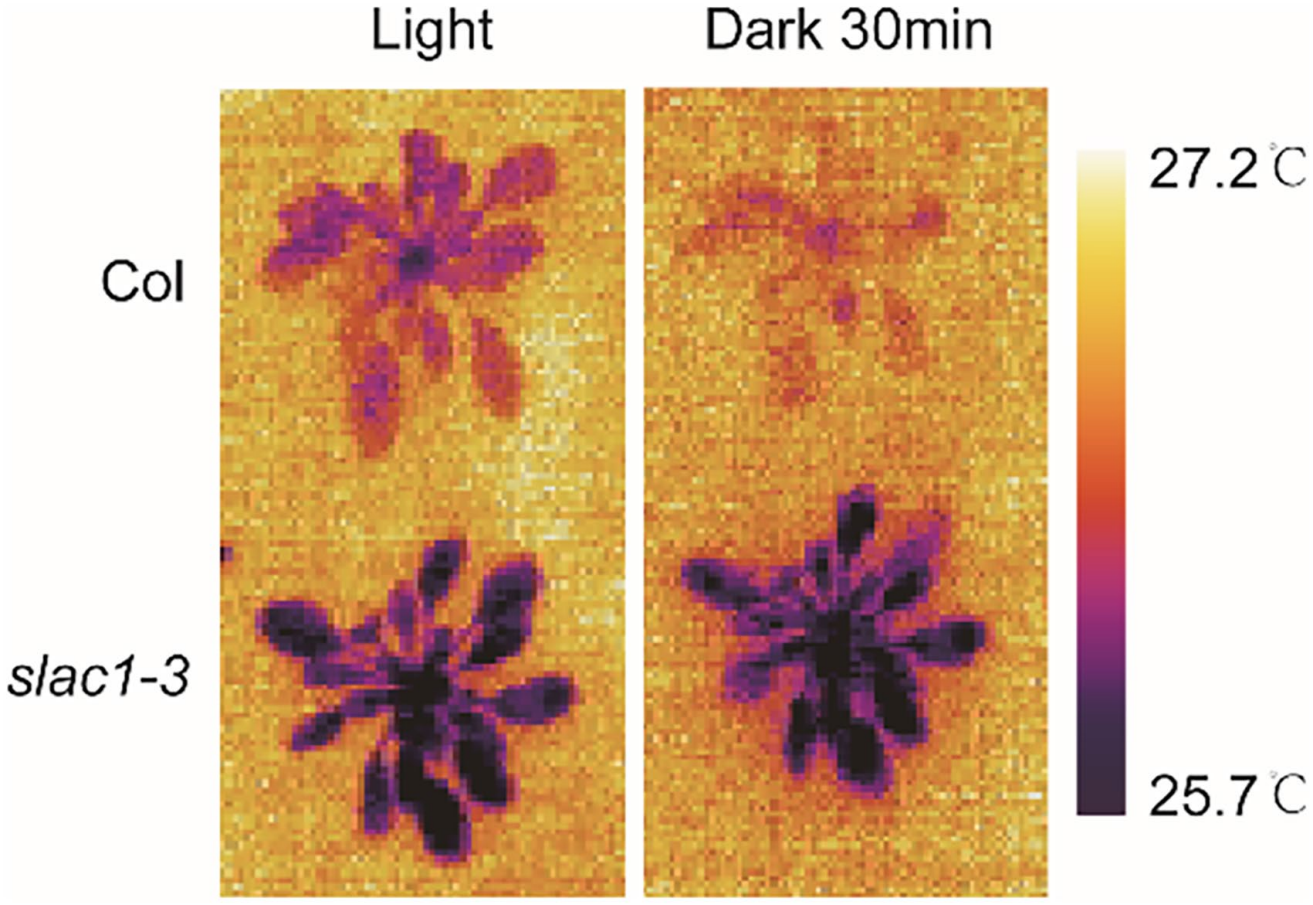
Determination of plant leaf temperature in Arabidopsis WT and *slac1-3* mutants in light and dark conditions. Multiple images of both genotypes under light/dark conditions were captured with thermal Imager. The representative images are shown here.

After establishing the defects in stimuli-induced stomatal closure, next, we aimed to confirm if this phenotype had any effect on leaf temperature, by using thermal imaging technique. Under light, *slac1-3* mutants showed lower leaf temperature compared with WT (Figure 2). After the dark treatment for 30 min, whereas the temperature of WT leaves clearly increased, the same was only slightly increased in mutants compared with its untreated counterpart (Figure 2). These data confirmed the defective dark-induced stomatal closure in *slac1-3* mutants.

### Transcriptome profiling identified 891 genes upregulated only in Arabidopsis *slac1-3* mutant under dehydration stress

**Figure 3:**
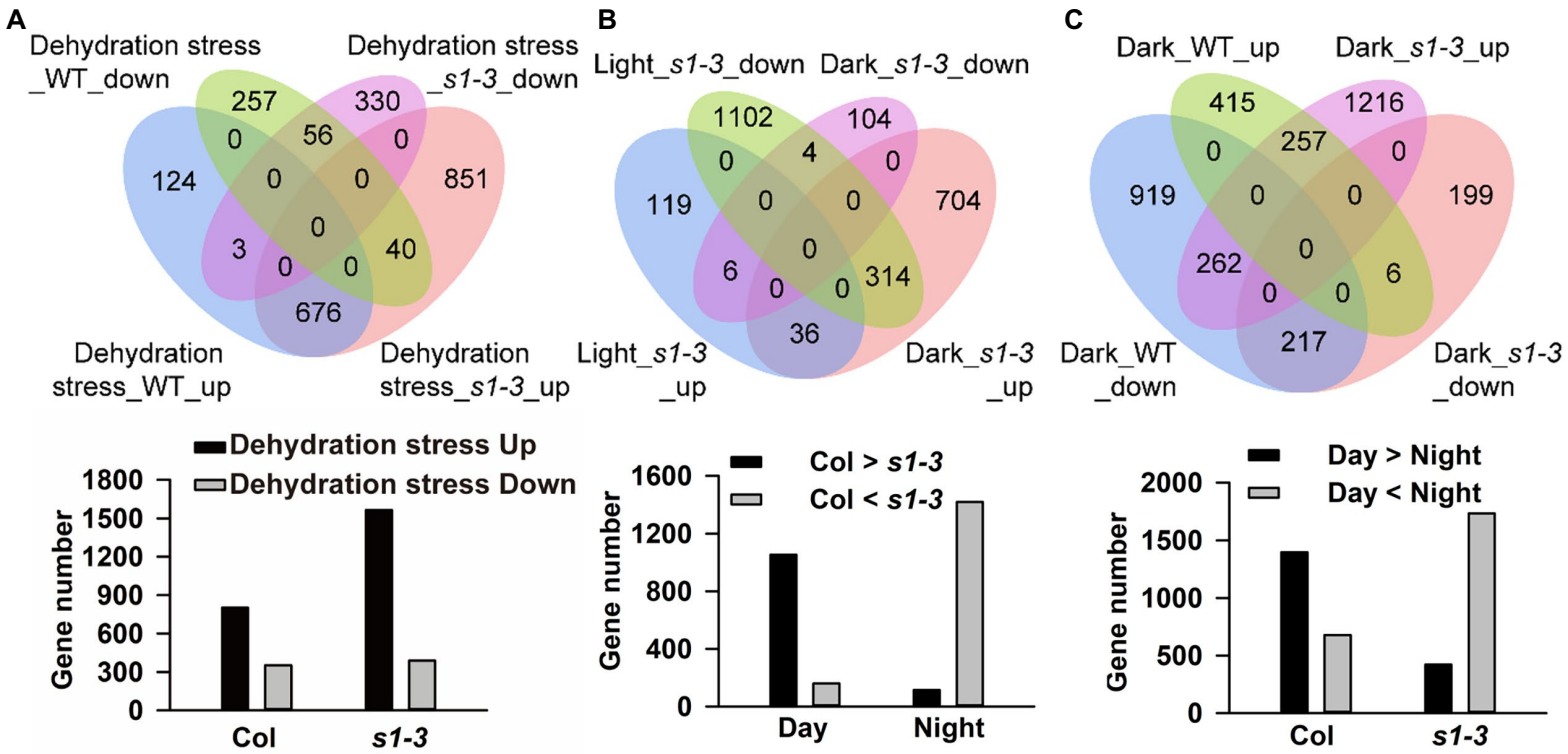
Differentially expressed gene (DEG) integration of each data set. DEGs of each data set are overlapped and presented as a Venn plot, including total, upregulated, and downregulated genes. DEGs, differently expressed genes. **(A)** Venn diagram of gene expression and gene number of up- or downregulated in dehydration stress; **(B)** up- or downregulated in dark conditions; and **(C)** up- or downregulated between WT and *slac1-3* mutant.

Next, we determined the molecular response of *slac1-3* mutant and WT in dehydration stress condition by carrying out global gene expression using RNA-sequencing. When exposed to dehydration for 25 min, *slac1-3* mutant showed highly variable response, compared to WT, with respect to changes in gene expression. Overall, the number of differentially expressed genes (DEGs; |log2 fold-change| ≥ 1 and value of *p* < 0.05) was much higher in *slac1-3* mutants than WT (1,956 vs. 1,156; Figure 3A). Interestingly, though the number of downregulated genes was not much different between mutant and WT (389 and 353), however, that of upregulated genes was much greater in *slac1-3* mutants than in WT (1,567 vs. 803). Of these, 891 genes were only upregulated in *slac1-3* mutants but not in WT in dehydration stress (Figure 3A).

**Figure 4:**
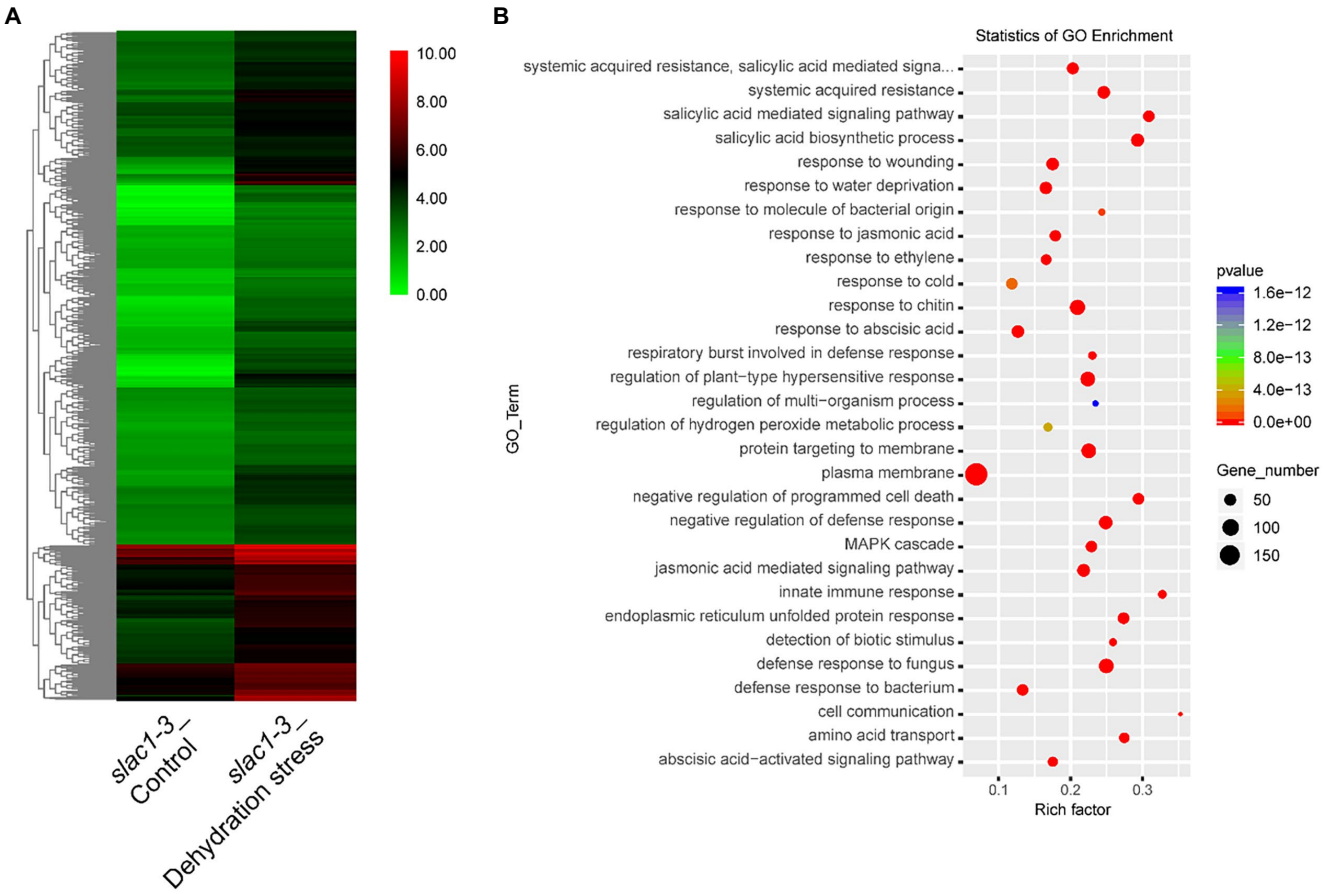
RNA-seq analysis of dehydration stress induced upregulated genes in *slac1-3* mutant. **(A)** Heat map of DEGs (891 in Figure 3A) in *slac1-3* mutants between control and dehydration stress **(B)** GO enrichment analysis of DEGs in *slac1-3* mutants in control and dehydration stress.

A heat map, representing the above mentioned 891 genes, was created based on FPKM of RNA-sequencing data to visualize the quantitative differences in the expression of these genes in *slac1-3* mutants under control and dehydration stress (Figure 4A). The level of expression of DEGs was divided into various classifications in the bar. The red and green regions are indicative of high and low expression, respectively. A color change from red to green, showing that the values of log10 (FPKM+1) change from high to low. Based on GO enrichment analysis the genes were classified into 30 functional categories (Figure 4B). The highest number of DEGs was related to plasma membrane function, followed by the genes related to plant hypersensitive response, response of chitin, defense response to fungus, protein targeting to membrane, response to water deficiency, and systemic acquired resistance (SAR). Based on enrichment factor, the major pathways significantly enriched in DEGs were cell communication pathway, innate immune response pathway, and salicylic acid biosynthesis and signaling pathways. To sum up, these data reveal the specific transcriptomic changes undertaken by the *slac1-3* mutant under dehydration stress to cope up with defective stomatal closure.

### Transcriptomic response of *slac1-3* mutant was variable than WT under light as well as dark

To determine the molecular response of two genotypes under day-night transition, we set to compare the pattern of gene expression in WT and mutants under light and dark. Under light conditions, 1,581 DEGs (161 upregulated and 1,420 downregulated) were identified between *slac1-3* mutant and WT (Figure 3B). On the other hand, 1,168 DEGs (1,054 upregulated and 114 downregulated) were identified between mutant and WT under dark conditions (Figure 3B). These findings point to a highly variable response of *slac1-3* mutant as compared with WT, under light as well as dark.

Further, we identified 314 DEGs which, compared with WT, had higher expression in mutant under dark but lower expression under light (Figure 3B).

Next, we set to get further insight into the transcriptome profiling of both genotypes under dark. The number of upregulated and downregulated genes was 678 and 1,398, respectively, in WT while it was 1,735 and 422, respectively, in *slac1-3* mutant under dark (Figure 3C). These data highlight the contrasting pattern of gene expression in the genotypes; under dark, whereas majority of DEGs in WT were found to be downregulated, in *slac1-3* majority were upregulated. Further, 262 DEGs were identified which were downregulated in WT but upregulated in mutant under dark. Since the dark-induced stomatal closure was defective in *slac1-3* mutants, the higher number of upregulated DEGs could represent a compensatory response of the mutant.

**Figure 5:**
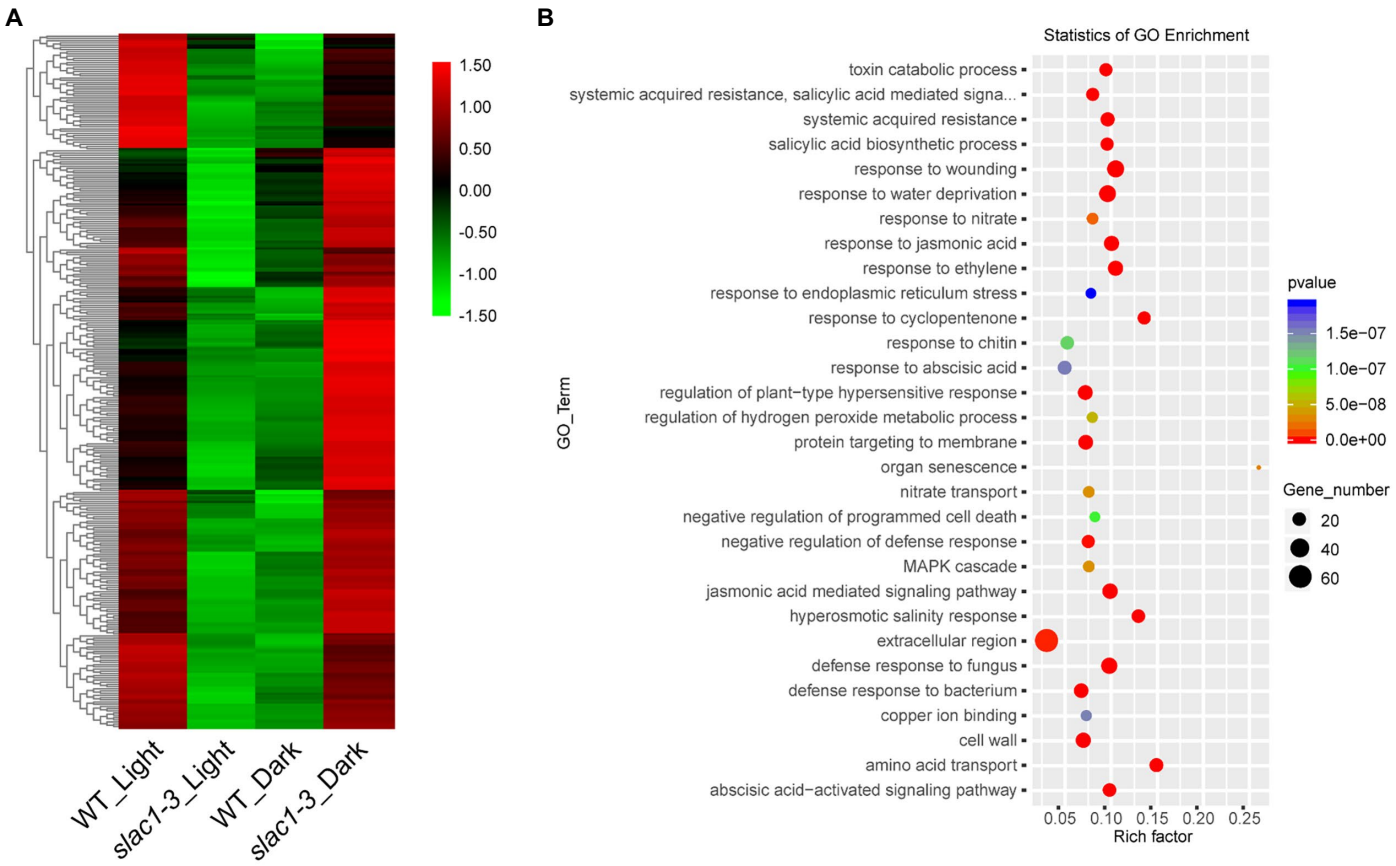
RNA-seq analysis of DEGs (314 in Figure 3B) at daytime or night. **(A)** Heat map of DEGs in *slac1-3* mutants in dark and light conditions **(B)** GO enrichment analysis of DEGs in *slac1-3* mutants in dark and light conditions.

We created a heat map of 314 DEGs shown in Figure 3B, to show the quantitative differences in the DEGs (Figure 5A). At daytime, these DEGs were downregulated in *slac1-3* mutant while, under dark, these were upregulated in the mutant, compared with WT (Figure 5A).

GO enrichment analysis was performed, and the genes were assorted into 30 functional categories (Figure 5B). The highest number of DEGs was related to extracellular region, wounding response, dehydration stress response, jasmonic acid and ethylene signaling pathways, and defense response to fungus. The major pathways significantly enriched with DEGs included the one associated with organ senescence, amino acid transport, hyperosmotic salinity response, and response to cyclopentenone.

**Figure 6:**
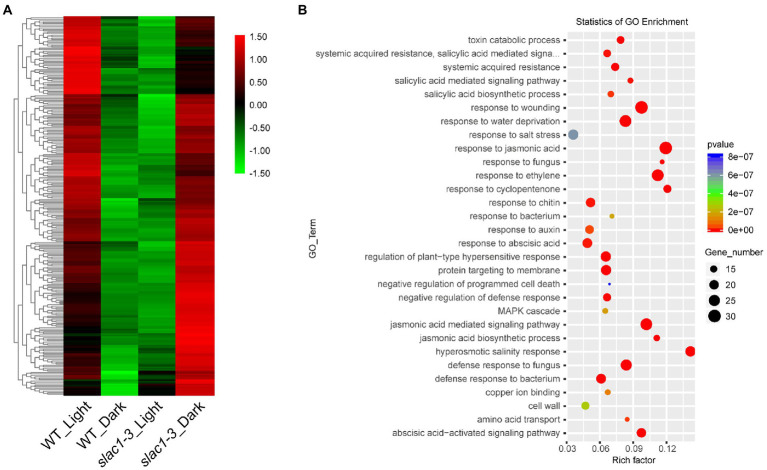
RNA-seq analysis of DEGs (262 in Figure 3C) between day and night. **(A)** Heat map of DEGs from day to night between *slac1-3* mutant and WT. **(B)** GO enrichment analysis of DEGs from day to night between *slac1-3* mutant and WT.

Next, we focused on the 262 DEGs (shown in Figure 3C) with opposite expression pattern in day/night between WT/mutant and developed a heatmap to represent quantitative differences in expression pattern (Figure 6A). These DEGs exhibited downregulation in WT but upregulation in *slac1-3* mutant under dark while in light these DEGs were up-regulated in WT but down-regulated in *slac1-3*. The DEGs were further subjected to GO enrichment analysis, and the genes were assigned a particular functional category (Figure 6B). Among these, DEGs belonging to hormonal response (jasmonic acid, ethylene, and abscisic acid), wounding response, defense to fungal disease, salt stress response, and jasmonic acid signaling pathway were over-represented. The major pathways significantly enriched with DEGs were related to hyperosmotic salinity response, and responses to cyclopentenone, jasmonic acid, ethylene, and fungus infection.

### Analysis of DEGs under light, dark, and dehydration stress

We further analyzed the DEGs shown in Figures 3A–C. The focus was on 40 DEGs upregulated in *slac1-3* mutant under dehydration stress but downregulated in WT (Figure 3A), 314 DEGs downregulated in *slac1-3* mutant under light, but upregulated in the mutant under dark, and 262 DEGs (shown in Figure 3C) with opposite expression pattern in day/night between WT/mutant. Table 1 shows a list of 18 unique DEGs with opposite expression pattern in *slac1-3* mutant and WT, under the above-mentioned conditions. These DEGs include drought-induced 21 (DI21), senescence related gene (SRG1), nitrilase 2 (NIT2), Kunitz trypsin inhibitor 1, plant invertase/pectin methyl esterase inhibitor superfamily, and lactoylglutathione lyase/glyoxalase I family protein.

**Table 1:** List of 18 DEGs common between dehydration stress (40, Figure 3A), *slac1-3* mutant (314, Figure 3B), and day and night (262, Figure 3C) showing opposite expression pattern in WT and mutant under different conditions.

**Gene ID**	**Light_Col *VS s1-3***	**Dark_Col *VS s1-3***	**Col_Light *VS* Dark**	***s1-3*_light *VS* Dark**	**Col_control *vs* Dehydration Stress**	***s1-3*_ control *VS* Dehydration Stress**	**Description**
AT1G62480	−4.74	1.32	−4.21	1.86	−3.52	1.55	Vacuolar calcium-binding protein-like protein
AT4G15910	−3.54	1.36	−3.19	1.71	−2.56	1.24	Drought-induced 21 (DI21)
AT4G37520	−4.01	1.39	−1.76	3.63	−3.72	1.26	Peroxidase superfamily protein
AT5G40730	−2.18	1.90	−2.77	1.31	−2.10	1.20	Arabinogalactan protein 24 (AGP24)
AT2G05540	−4.93	2.01	−3.75	3.19	−2.27	3.28	Glycine-rich protein family
AT5G07440	−2.28	2.35	−1.94	2.69	−1.53	1.66	Glutamate dehydrogenase 2 (GDH2)
AT4G15610	−4.93	2.53	−3.04	4.41	−3.10	3.23	Uncharacterized protein family (UPF0497)
AT1G76930	−3.20	2.94	−3.57	2.57	−2.87	2.47	Extensin 4 (EXT4)
AT1G17020	−4.33	3.12	−2.50	4.95	−2.95	4.09	Senescence-related gene 1 (SRG1)
AT1G23800	−2.40	3.21	−1.91	3.69	−2.57	3.14	Aldehyde dehydrogenase 2B7 (ALDH2B7)
AT1G74010	−3.59	3.29	−2.17	4.71	−2.53	3.69	Calcium-dependent phosphotriesterase superfamily protein
AT2G29350	−4.64	3.53	−2.62	5.56	−2.00	4.07	Senescence-associated gene 13 (SAG13)
AT5G54510	−5.44	3.58	−2.99	6.03	−3.98	4.41	Auxin-responsive GH3 family protein (DFL1)
AT4G33150	−3.45	3.63	−2.48	4.61	−1.90	2.15	lysine-ketoglutarate reductase/saccharopine dehydrogenase bifunctional enzyme
AT3G44300	−5.14	3.94	−3.65	5.43	−4.48	3.93	Nitrilase 2 (NIT2)
AT1G73260	−10.62	4.40	−3.99	11.03	−4.00	7.66	Kunitz trypsin inhibitor 1 (KTI1)
AT2G45220	−5.37	5.05	−4.61	5.80	−2.90	3.81	Plant invertase/pectin methylesterase inhibitor superfamily (GLYI7)
AT1G80160	−6.18	5.25	−3.03	8.40	−3.44	6.19	Lactoylglutathione lyase / glyoxalase I family protein

The positive values represent upregulation while negative values represent downregulation.

Furthermore, in *slac1-3* mutants, six DEGs were identified showing higher expression in WT (and lower expression in *slac1-3* mutant) under light but lower expression in WT (and higher expression in *slac1-3* mutant) under dark (Table 2). These included RING/FYVE/PHD zinc finger superfamily protein, three hypothetical proteins, and a transmembrane protein.

**Table 2:** List of six DEGs (Figure 3B) showing higher expression in WT (and lower expression in *slac1-3* mutant) under light but lower expression in WT (and higher expression in *slac1-3* mutant) under dark.

**Gene ID**	**Light_Col *VS s1-3***	**Dark_Col *VS s1-3***	**Description**
AT5G63780	1.05	−1.16	RING/FYVE/PHD zinc finger superfamily protein (SHA1)
AT4G04293	1.14	−1.33	Hypothetical protein
AT5G03120	1.16	−1.87	Transmembrane protein
AT3G11110	1.19	−1.48	RING/U-box superfamily protein
AT5G19190	1.22	−1.27	Hypothetical protein
AT3G06070	1.47	−1.62	Hypothetical protein

The positive values represent upregulation while negative values represent downregulation.

As shown in Table 3, six DEGs were identified with opposite pattern of expression in WT and mutant under light–dark transition. These include an ovate family protein 16, a HXXXD-type acyl-transferase family protein, and a myo-inositol oxygenase 1 protein. We also identified three DEGs which, under dehydration stress, were upregulated in *slac1-3* mutant but downregulated in WT. One of these proteins was phloem protein 2-A13 while the other was nine-cis-epoxycarotenoid dioxygenase 4 (NCED4; Table 4).

**Table 3:** List of six DEGs (Figure 3C) with opposite expression pattern in mutant and WT in light–dark transition (in light upregulation in WT but in dark upregulation in *slac1-3* mutant).

**Gene ID**	**Col_Light *VS* Dark**	**s1-3_Light *VS* Dark**	**Description**
AT4G04293	1.07	−1.40	Hypothetical protein
AT5G19190	1.46	−1.03	Hypothetical protein
AT3G06070	1.39	−1.71	Hypothetical protein
AT2G32100	1.05	−2.01	Ovate family protein 16 (OFP16)
AT5G39080	1.01	−1.17	HXXXD-type acyl-transferase family protein
AT1G14520	1.18	−1.02	Myo-inositol oxygenase 1 (MIOX1)

The positive values represent upregulation while negative values represent downregulation.

**Table 4:** List of three DEGs (Figure 3A) with opposite expression pattern in mutant and WT (downregulated in WT but upregulated in *slac1-3* mutant under dehydration stress).

**Gene ID**	**Col_Control vs. Dehydration Stress**	***s1-3*_Control vs. Dehydration Stress**	**Description**
AT5G11070	1.46	−1.27	Hypothetical protein
AT3G61060	1.28	−1.03	Phloem protein 2-A13 (PP2-A13)
AT4G19170	1.19	−2.46	Nine-cis-epoxycarotenoid dioxygenase 4 (NCED4)

The positive values represent upregulation while negative values represent downregulation.

## Discussion

Owing to their central role in regulating CO_2_ uptake and transpiration in plants, changes in stomatal opening and development exhibit adaptive relationships to the environmental conditions in which the plant is growing (Richardson et al., 2017). Being sessile in nature, plants adjust their physiological processes like stomatal movements in response to external factors. The guard cells perceive and respond to various environmental cues to optimize the gas exchange, and at the same time avoiding drought stress (Pillitteri and Torii, 2012). Being a central player in mediating stimuli-induced stomatal closure, SLAC1 seems to be a converging point for different stomata closure pathways.

The regulation of stomatal movements could also be governed through transcriptional changes (Sirichandra et al., 2009) as demonstrated by the fact that the transcriptional inhibitors negatively affected stomatal opening, and that two RNA-binding proteins were involved in ABA-induced stomatal closure (Hugouvieux et al., 2001; Li et al., 2002). Furthermore, studies focusing on ABA-induced transcriptomic changes in the guard cell have led to the identification of transcription factors (TFs) involved in regulating the stomatal movements. The TFs involved in stomatal closure include AtMYB15, AtMYB44, AtMYB61, and SNAC1 etc. (Cominelli et al., 2010). Hence it seems that gene expression also plays important role in stomatal movements.

It is evident that failure in proper stomatal closure and/or opening could lead to changes in gene expression. We identified many differentially modulated genes between *slac1-3* mutant and WT under different stimuli like light, dark, and dehydration stress (Figures 3A–C), showing that mutation in SLAC1 results in larger rearrangements at transcriptome level. So, what could be the link between SLAC1 disruption and alterations in gene expression? The failure of stomata to close normally results in water loss, changes in leaf temperature, osmotic changes, and pathogen invasion etc. All these factors can affect the expression of many genes. Moreover, the altered gene expression could be plant’s compensatory response due the failure in properly opening and closing the stomatal pore. It is already known that aberrations in stomatal movements lead to compensatory alterations in gene expression, or reorganization of the signaling pathways to compensate for the WT function of the gene (Webb and Baker, 2002).

It is well established that stomatal movements affect the transpiration rate which results in changes in osmotic potential and leaf temperature (Kaňa and Vass, 2008). Similarly, stomatal movements induced by day-night transition also result in changes in osmotic potential and temperature. As shown in Figure 2, the leaves of *slac1-3* mutants were cooler than WT in dark, which could have led to differences in the expression pattern of genes between WT and the mutant. These data showed that stomatal switch could be linked to changes in gene expression. Moreover, it has been demonstrated that guard cells of *slac1-3* mutants exhibit higher resting Ca^2+^ level (Laanemets et al., 2013). As Ca^2+^ is a ubiquitous signaling molecule, therefore, perturbations in Ca^2+^ level could also be associated with differential gene expression in *slac1-3* mutants.

When challenged with drought stress, plants adopt few urgent measures alongside few long-term responses. The former, known as drought avoidance response, includes immediate stomatal closure to conserve water (Dobra et al., 2010) while the long-term response includes induction of drought stress responsive genes. As demonstrated in our study, dehydration stress-induced stomatal closure was defective in *slac1-3* mutants (Figure 1) and therefore, these mutants lost more water than WT from detached leaves. As the extent of dehydration stress was more pronounced in mutants, therefore, a significantly higher number of genes were upregulated in dehydration stress only in the mutant but not in WT, possibly to initiate compensatory responses.

We identified few DEGs in mutant which could represent such compensatory mechanisms. For example, a gene encoding for nine-cis-epoxycarotenoid dioxygenase 4 (NCED4) was about 2.5-fold upregulated in *slac1-3* mutant under dehydration stress (Table 4). NCED is involved in *de novo* ABA synthesis and catalyzes the first committed step in synthesizing ABA by converting 9-cis-neoxanthin to xanthoxin (Tan et al., 1997; Huang et al., 2018). It was observed that detached leaves of *slac1* mutants lost water significantly faster than WT, showing defects in transpiration regulation under drought stress (Negi et al., 2008; Vahisalu et al., 2008). Since the mutant is unable to properly close stomata under dark and dehydration stress, the upregulation of NCED could reflect a strategy by *slac1-3* mutant to promote ABA biosynthesis and thus induce stomatal closure to avoid water loss. Similarly, another gene encoding a member of ovate family had two-fold higher expression in *slac1-3* mutants than WT (Table 3). Ovate family proteins (OFPs) are plant-specific transcription factors, which regulate the content of epicuticular wax, a coating of wax covering the outer surface of the plant cuticle in land plants to reduce the water loss *via* surfaces (Tang et al., 2018). It is possible that lack of normal stomatal closure, could be linked with deploying other strategies like increasing epicuticular waxes for reducing water loss from plant surface. However, further investigations will be needed to experimentally prove if mutant deposit more wax over their surfaces.

In current study, we found that a lactoylglutathione lyase family protein (also known as Glyoxalase I) had lower expression in *slac1-3* mutants in dark and dehydration stress (Table 1). Under abiotic stress, methylglyoxal (MG), a toxic molecule, accumulates in plants. It has been reported that at a certain optimum concentration MG induces stomatal closure, involving oxidative burst and [Ca^2+^]cyt oscillations but in ABA-independent manner (Hoque et al., 2012a,b). MG also inhibits Kin channel currents in Arabidopsis guard cells and interferes with light-induced stomatal opening (Hoque et al., 2012a,b, 2016). Glyoxalase I (also known as lactoylglutathione lyase) is involved in MG detoxification (Norton et al., 1990; Maiti et al., 1997). It seems that by keeping glyoxalase I expression at low level, mutant could be attempting to increase MG level and thereby promote stomatal closure under dehydration stress and darkness. However, further experimentation is needed to prove whether differential expression of Glyoxalase I had any impact on MG level by directly determining the latter’s level in both genotypes.

Interestingly, under both stimuli for stomatal closure (i.e., dark and dehydration stress), a major chunk of DEGs was found to be associated with abiotic and biotic stress responses (e.g., response to bacterial and fungal infections, wounding, chilling, salinity, dehydration stress etc.) and stress related-hormonal pathways (e.g., abscisic acid, salicylic acid, jasmonic acid, and ethylene pathways). This shows that defects in stimulus-induced stomatal closure may result in the induction of stress response pathways.

**Figure 7:**
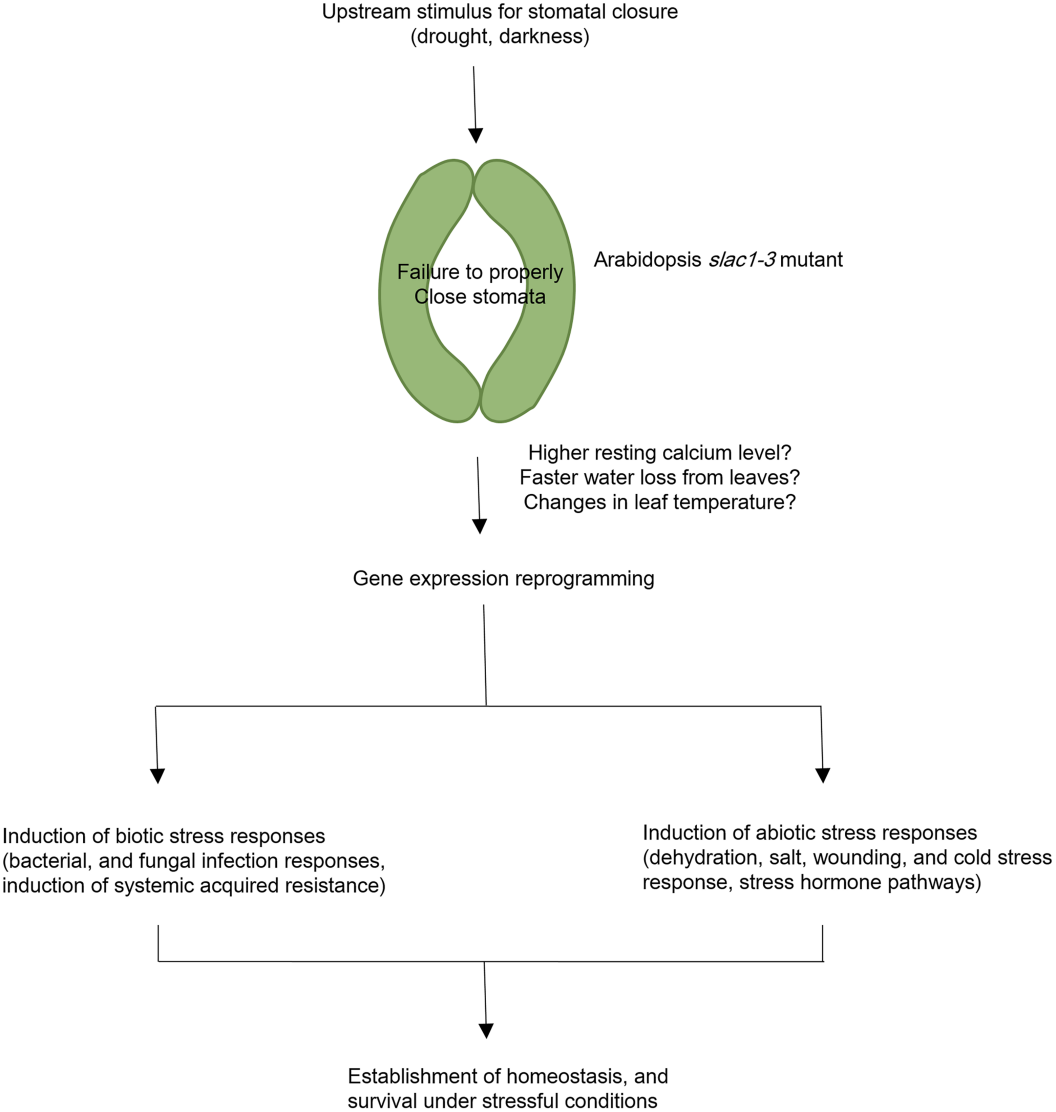
A model representing transcriptomic changes undertaken by Arabidopsis *slac1-3* mutant in response to dehydration stress and dark treatment. Under these stimuli, *slac1-3* mutant shows defective stomatal closure. Resultantly, plants lose more water and exhibit cooler leaf temperature. Mutation in Arabidopsis SLAC1 is also linked with higher resting calcium level in mutant. These factors could be among the possible links between *slac1-3* mutation and differential transcriptomic response in mutant. To compensate for defective stomatal closure and its consequences, mutant undergo biotic and abiotic stress responses. The former involves induction of bacterial and fungal infection pathways, and systemic acquired resistance while the latter involves dehydration, salt, wounding, and cold stress responses, and induction of stress hormone pathways. However, it should be noted that few of these hormones may also be simultaneously associated with biotic stress response.

To conclude, in current study, we have identified specific changes in gene expression underlying *slac1-3* mutation and resultant defects in stomatal closure in Arabidopsis. The large-scale changes in gene expression in *slac1-3* mutants could be attributed to changes in osmotic potential, leaf temperature, and/or higher resting Ca^2+^ level in mutant. Furthermore, the induction of stress responsive pathways in mutant could be suggestive of the role of stomata as a switch in triggering abiotic and biotic stress responsive genes, which could help the plant to survive under stressful conditions. The conclusions of current research are presented as a model in Figure 7.

## Data availability statement

The original contributions presented in the study are included in the article/Supplementary material, further inquiries can be directed to the corresponding authors.

## Author contributions

ZW, SW, DX, and YX: methodology, formal analysis, investigation, and writing–review and editing. YO: methodology, formal analysis, investigation, and writing–original draft. HR: conceptualization, methodology, formal analysis, investigation, writing–original draft, writing–review and editing, and funding acquisition. JH: conceptualization and manuscript writing, review, and editing. GQ: conceptualization. All authors contributed to the article and approved the submitted version.

## Funding

This study was supported by the Natural Science Foundation of Zhejiang Province (Grant No: LY20C020001), the Open Project Program of State Key Laboratory of Crop Stress Biology for Arid Areas, NWFU, Yangling, Shanxi, China (Grant No: CSBAAKF2021008), and the State Key Laboratory of Subtropical Silviculture (Grant No: ZY20190201).

## Conflict of interest

The authors declare that the research was conducted in the absence of any commercial or financial relationships that could be construed as a potential conflict of interest.

## Publisher’s note

All claims expressed in this article are solely those of the authors and do not necessarily represent those of their affiliated organizations, or those of the publisher, the editors and the reviewers. Any product that may be evaluated in this article, or claim that may be made by its manufacturer, is not guaranteed or endorsed by the publisher.

## Supplementary material

The Supplementary material for this article can be found online at: https://www.frontiersin.org/articles/10.3389/fpls.2022.987606/full#supplementary-material

Supplementary Figure S1 Venn diagram of DEGs between dehydration stressdrought (40, Figure 3A), slac1-3 mutant 698 (314, Figure 3B), and day and night (262, Figure 3C).

## Footnotes

^1^ www.omicshare.com/tools
