# Supplementary figures and images for: Transcriptome profiling of Arabidopsis slac1-3 mutant reveals compensatory alterations in gene expression underlying defective stomatal closure
Source: Front Plant Sci. 2022 Sep 20;13:987606. doi: 10.3389/fpls.2022.987606 (PMC9530288; doi:10.3389/fpls.2022.987606)

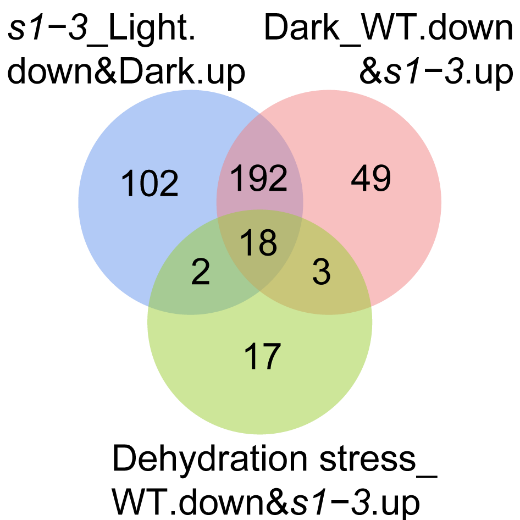

Supplement: Supplementary Figure S1 — Venn diagram of DEGs between dehydration stressdrought (40, Figure 3A), slac1-3 mutant 698 (314, Figure 3B), and day and night (262, Figure 3C). [file Image_1.TIF]
